# Evaluation of machine learning algorithms and structural features for optimal MRI-based diagnostic prediction in psychosis

**DOI:** 10.1371/journal.pone.0175683

**Published:** 2017-04-20

**Authors:** Raymond Salvador, Joaquim Radua, Erick J. Canales-Rodríguez, Aleix Solanes, Salvador Sarró, José M. Goikolea, Alicia Valiente, Gemma C. Monté, María del Carmen Natividad, Amalia Guerrero-Pedraza, Noemí Moro, Paloma Fernández-Corcuera, Benedikt L. Amann, Teresa Maristany, Eduard Vieta, Peter J. McKenna, Edith Pomarol-Clotet

**Affiliations:** 1 FIDMAG - Germanes Hospitalaries, Barcelona, Spain; 2 Centro de Investigación Biomedica en Red de Salud Mental (CIBERSAM), Barcelona, Spain; 3 Institute of Psychiatry, Psychology and Neuroscience, King’s College, London, United Kingdom; 4 Center for Psychiatry Research, Department of Clinical Neuroscience, Karolinska Institutet, Stockholm, Sweden; 5 University of Barcelona, Barcelona, Spain; 6 Hospital Clínic, University of Barcelona, IDIBAPS, Barcelona, Spain; 7 Hospital Benito Menni – CASM, Sant Boi de Llobregat, Spain; 8 Hospital Mare de Déu de la Mercè, Barcelona, Spain; 9 Institut de Neuropsiquiatria i Addiccions, Centre Fòrum Research Unit, Parc de Salut Mar, Barcelona, Spain; 10 IMIM (Hospital del Mar Medical Research Institute), Barcelona, Spain; 11 Department of Psychiatry, Autonomous University of Barcelona, Barcelona, Spain; 12 Hospital Sant Joan de Déu, Esplugues de Llobregat, Spain; National University of Defense Technology College of Mechatronic Engineering and Automation, CHINA

## Abstract

A relatively large number of studies have investigated the power of structural magnetic resonance imaging (sMRI) data to discriminate patients with schizophrenia from healthy controls. However, very few of them have also included patients with bipolar disorder, allowing the clinically relevant discrimination between both psychotic diagnostics. To assess the efficacy of sMRI data for diagnostic prediction in psychosis we objectively evaluated the discriminative power of a wide range of commonly used machine learning algorithms (ridge, lasso, elastic net and L0 norm regularized logistic regressions, a support vector classifier, regularized discriminant analysis, random forests and a Gaussian process classifier) on main sMRI features including grey and white matter voxel-based morphometry (VBM), vertex-based cortical thickness and volume, region of interest volumetric measures and wavelet-based morphometry (WBM) maps. All possible combinations of algorithms and data features were considered in pairwise classifications of matched samples of healthy controls (N = 127), patients with schizophrenia (N = 128) and patients with bipolar disorder (N = 128). Results show that the selection of feature type is important, with grey matter VBM (without data reduction) delivering the best diagnostic prediction rates (averaging over classifiers: schizophrenia vs. healthy 75%, bipolar disorder vs. healthy 63% and schizophrenia vs. bipolar disorder 62%) whereas algorithms usually yielded very similar results. Indeed, those grey matter VBM accuracy rates were not even improved by combining all feature types in a single prediction model. Further multi-class classifications considering the three groups simultaneously made evident a lack of predictive power for the bipolar group, probably due to its intermediate anatomical features, located between those observed in healthy controls and those found in patients with schizophrenia. Finally, we provide MRIPredict (https://www.nitrc.org/projects/mripredict/), a free tool for SPM, FSL and R, to easily carry out voxelwise predictions based on VBM images.

## Introduction

Although the role of statistical methods in medical research has been historically dominated by inference, its use for prediction has become more relevant in recent years. In part, this shift in objectives has been allowed by the availability of large amounts of data together with the development of new computational tools that can deal with these large datasets [1]. Among other sources, structural magnetic resonance imaging (sMRI) data has been proposed as an input for clinical diagnosis and outcome prediction in different clinical areas [2].

Initially, due to the large extent of MRI datasets, intermediate steps aimed at reducing the number of predictor variables were required for computational feasibility. Such reduction could either involve a supervised step, where the researcher selected specific voxels or brain regions based on a priori information (i.e. feature selection), or an unsupervised procedure like a principal or independent component analysis [3]. In both cases, though, the risk of discarding relevant information was present. In recent years, however, optimized versions of commonly used classifiers which can be readily applied to MRI datasets without needing dimensionality reduction have been developed [4].

Studies evaluating the predictive power of sMRI images are particularly numerous in Alzheimer’s disease prediction [5], psychiatric diagnosis [6, 7] and in the assessment of brain tumor characteristics [8]. Still, it is difficult to extract reliable conclusions on optimal prediction procedures from individual studies as they usually evaluate the performance of specific algorithms on image sets that have been acquired and processed in particular ways, with only a small subset of studies systematically comparing the prediction capacity of available algorithms. While this comparison has been recently made for several pathologies including multiple sclerosis [9], fibromyalgia [10] and Alzheimer’s disease [11, 12] some other relevant clinical areas such as psychosis still lack a systematic evaluation.

Specifically, in the area of psychosis, where studies have traditionally focused on reporting statistically significant differences involving patients with schizophrenia and patients with bipolar disorder, there is a current interest in predicting the final diagnostic for patients undergoing a psychotic episode by means of these classifying algorithms. Most of the sMRI studies carried out so far, though, have mainly assessed the classification accuracy between patients with schizophrenia and controls [7], with only few evaluating the discriminative power of sMRI to separate patients with bipolar disorder from healthy subjects [13–16] and only one of them performing the most clinically relevant classification between bipolar and schizophrenic subjects [14].

Here, in order to objectively assess the utility of sMRI images in diagnostic prediction in psychosis, we systematically evaluate the performance of a large set of available machine learning algorithms (ridge, lasso, elastic net and L0 norm regularized logistic regressions, a support vector classifier, regularized discriminant analysis, random forests and a Gaussian process classifier) on some of the most commonly used sMRI data formats (grey and white matter voxel-based morphometry, vertex-based cortical thickness and volume, region of interest volumetric measures and wavelet-based morphometry maps). All possible combinations of algorithms and data formats are used to estimate the discriminability between well matched samples of healthy controls (N = 127), of patients with schizophrenia (N = 128) and of patients with bipolar disorder (N = 128). Furthermore, to maximize the predictive power of sMRI images, all different feature types are also combined in a single prediction model. Finally, several multi-class approaches are considered in order to evaluate the accuracy rates to be found in a simultaneous classification of the three groups. As detailed later, we provide as well MRIPredict, a free tool for SPM, FSL and R that allows an easy specification, validation and fitting of voxelwise models that can be later applied to new MRI datasets, even if they have different voxel dimensions (software available at https://www.nitrc.org/projects/mripredict/).

## Material and methods

### Sample

A sample of N = 128 individuals with a diagnosis of schizophrenia according to DSM-IV criteria were recruited from Benito Menni CASM and Mare de Déu de la Mercè hospitals (Spain). All individuals were right handed, in the 18 to 65 age interval, with no history of brain trauma or neurological disease, and not having shown alcohol/substance abuse in the last 12 months. All patients but one were taking antipsychotic medication (atypical N = 82, typical N = 9, both N = 30, unknown N = 6, equivalents of Chlorpromazine: 824.0 mg (mean), 642.8 mg (sd)). Considering the same exclusion criteria, a second sample of N = 128 patients with a diagnose of type I bipolar disorder matched for age, gender and pre-morbid IQ, as estimated with the Word Accentuation Test [17] were recruited from the Benito Menni CASM and the Hospital Clínic de Barcelona (Spain). When scanned, 77 were in euthymia while 28 were undergoing a manic phase and 23 were under depression. 75 where taking antipsychotic medication (atypical N = 64, typical N = 4, both N = 7, equivalents of Chlorpromazine: 399.4 mg (mean), 388.0 mg (sd)), 105 where taking mood stabilizers and 33 antidepressants. Finally, a third sample of N = 127 healthy control individuals, matched by the same criteria was recruited from non-medical hospital staff, their relatives and acquaintances, plus independent sources in the community. Apart from previous exclusion criteria, controls reporting a history of mental illness and/or treatment with psychotropic medication were discarded. Table 1 gives further demographic and clinical information on the three samples. All participants gave written informed consent and the study was approved by the Clinical Research Ethics Committee of the Sisters Hospitallers (Comité de Ética de Investigación Clínica de las Hermanas Hospitalarias).

**Table 1:** Demographic and clinical characteristics of samples of both patient groups and of healthy controls.

	Schizophrenia N = 128	Bipolar disorder N = 128	Controls N = 127	Statistical significance
Age	41.5 (10.3) Range: 18–65	41.4 (10.4) Range: 20–64	39.8 (10.3) Range: 20–64	p(sch, bip) = 0.94 p(sch, cnt) = 0.18 p(bip, cnt) = 0.21
Gender (F/M)	54/74	54/74	54/73	p(sch, bip) = 1.0 p(sch, cnt) = 0.96 p(bip, cnt) = 0.96
Illness duration	18.4 (11.0) Range: 0–43	14.7 (10.6) Range: 0–42		p(sch, bip) = 0.0084
^a^TAP	22.09 (4.85)	22.72 (4.31)	23.0 (4.41)	p(sch, bip) = 0.29 p(sch, cnt) = 0.14 p(bip, cnt) = 0.64
^b^WAIS-III	91.9 (17.6)	93.4 (15.6)	107.2 (15.5)	p(sch, bip) = 0.51 p(sch, cnt)< 0.0001 p(bip, cnt)< 0.0001
^c^PANSS	72.6 (17.5)	46.0 (16.1)		p(sch,bip)< 0.0001
PANSS positive	16.9 (5.7)	10.4 (5.5)		p(sch,bip)< 0.0001
PANSS negative	21.4 (7.0)	10.7 (5.3)		p(sch,bip)< 0.0001
PANSS Gen. Psych.	34.3 (8.4)	24.9 (9.9)		p(sch,bip)< 0.0001
^d^YMRS		5.95 (9.72)		
^e^HDRS		7.43 (9.34)		

^a^TAP: Word Accentuation Test (Test de Acentuación de Palabras);

^b^WAIS-III: Wechsler Adult Intelligence Scale III;

^c^PANSS: Positive and Negative Syndrome Scale;

^d^YMRS: Young Manic Rating Scale;

^e^HDRS: Hamilton Depression Rating Scale.

Values given for single groups are mean and standard deviations.

### sMRI data features

**Fig 1:**
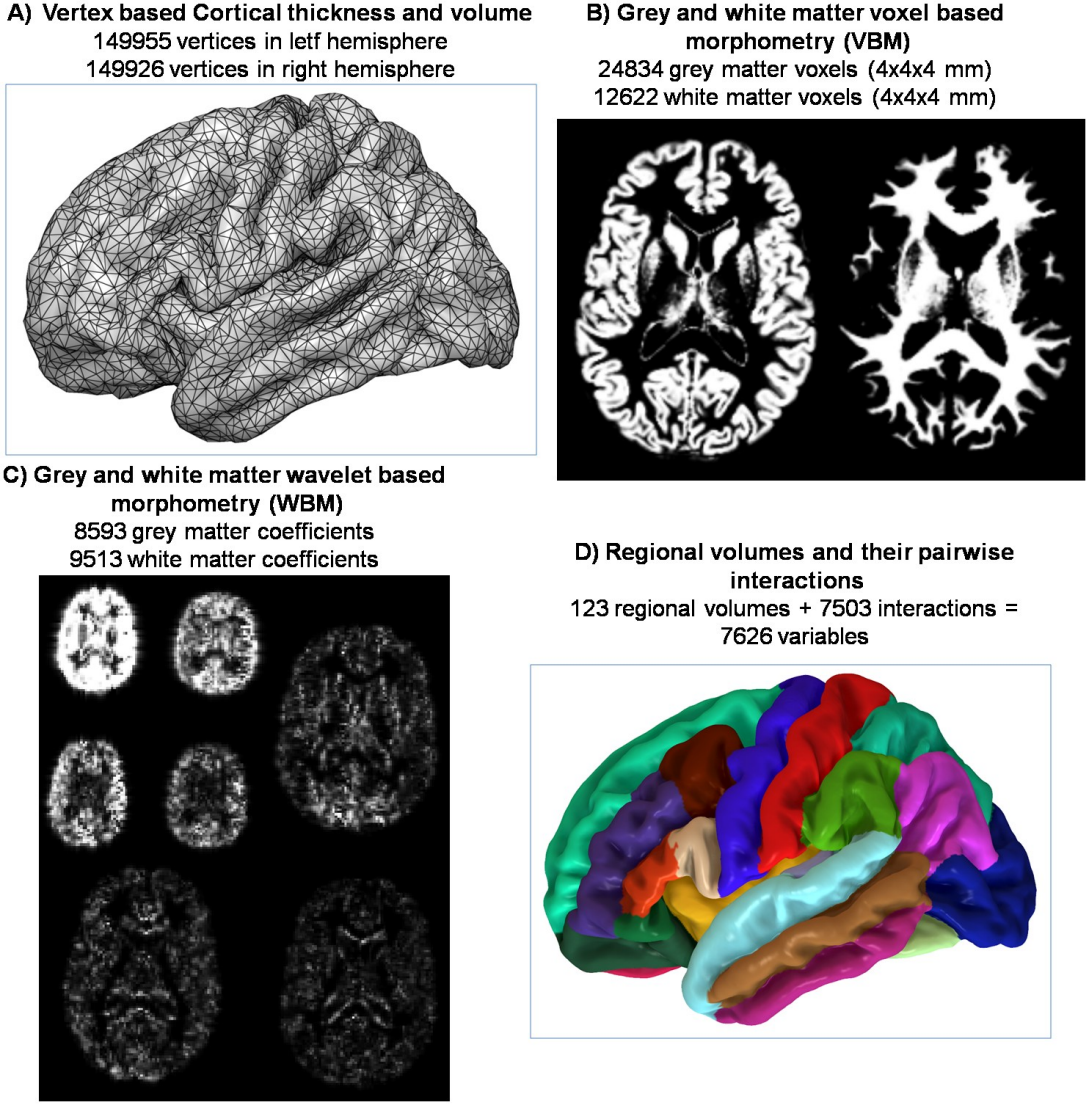
Data features generated from the individual structural T1 magnetic resonance images. Each type was used as input data to evaluate the prediction capacity of the different classifiers. Grey and white matter was considered separately when using voxel based features. Left and right hemispheres were considered separately when vertex based cortical information was applied.

For each subject, a structural brain image was acquired with a 1.5-T GE Signa scanner (General Electric Medical Systems, Milwaukee, WI, USA) using the following acquisition parameters: T1-weighted sequence, 180 axial slices, 1mm slice thickness with no gap, 512×512 matrix size, 0.5×0.5×1mm^3^ voxel resolution, 4ms echo time, 2000ms repetition time, 15° flip angle. Once acquired, information contained in the T1 images was summarized in the following data features (see also Fig 1):

1. Cortical thickness of left and right hemispheres: sMRI data were analyzed with the FreeSurfer image analysis suite (http://surfer.nmr.mgh.harvard.edu/). Briefly, the pre-processing included removal of non-brain tissue, automated Talairach transformation, tessellation of the grey and white matter boundaries and surface deformation [18]. A number of deformation procedures were performed in the data analysis pipeline, including surface inflation and registration to a spherical atlas. Intensity and continuity information from the entire three dimensional images in the segmentation and deformation procedures were used to produce vertex-wise representations of cortical thickness (CT) in each vertex across the cortical mantle.
2. Cortical volume of left and right hemispheres: In addition to CT, the FreeSurfer also computes vertex-wise cortical surface area (SA). Both CT and SA are multiplied to obtain a vertex-wise representation of cortical volume (CV). All individual CT and CV maps were smoothed using a Gaussian kernel with full width at half maximum (FWHM) of 30 mm [19].
3. Grey and White matter voxel based morphometry (VBM) images: Structural images were segmented into grey and white matter partial volume images in the native space using the unified segmentation algorithm included in SPM12 [20]. Then, the original structural images were brain-extracted [21] and aligned to the Montreal Neurological Institute MNI152 2mm standard template using FSL registration tools [22]. The resulting deformation fields were applied to the initially segmented images to obtain grey and white matter normalized images. To reduce computational cost, those images were subsampled to a 4 x 4 x 4 mm resolution.
4. Grey and White matter wavelet based morphometry (WBM) images: Taking the grey and white matter normalized VBM images as inputs, we applied the methodology explained in [23] and implemented in the WBM toolbox (http://www.wbmorphometry.com/). Initially, input images were smoothed with a Gaussian kernel (FWHM≈7 mm) and were transformed to the wavelet-domain using a 3D discrete orthogonal wavelet transform based on symmetric spline wavelets with degree n = 3 and resolution level J = 2. By means of the minimum description length procedure, coefficients that best represented grey and white matter anatomy on all subjects were retained for the classifications.
5. Region of interest (ROI) based brain volumes and their interactions: The FreeSurfer was used to parcellate the brain parenchyma in cortical and subcortical ROIs [24]. Mean volume values for these ROIs were extracted and used together with cerebellum, white matter and ventricle volumes as independent variables for classification. In addition, after standardizing their values, we calculated the pairwise products between all volumes as modelers of pairwise interaction. This extended set of variables was also supplied to the classifiers together with the original regional volumes.
6. Joint dataset combining all previous data features: We evaluated the potential improvement in classification accuracy achieved by merging data from all feature types in a single matrix. The amount of independent variables involved, however, made the direct implementation of algorithms computationally unfeasible. To reduce the number of variables in a meaningful way we implemented two different strategies: (a) following Wang et al. [25], we applied a previous dimensionality reduction through a principal component analysis (PCA), and (b) by considering a similar approach than in Dai et al. [26], we calculated the univariate t-values from pairwise group comparisons, selecting only the 1% of variables with highest t-scores (in absolute value).

### Learning algorithms

Eight classifiers, selected for their habitual usage and their computational efficiency, were applied to the different data features described in the previous section. Prediction capability of each data feature—classifier pair on the three possible classifications involving the two groups of patients and controls was quantified. Specifically, the algorithms evaluated were: (I) ridge and (II) lasso logistic regressions [27], (III) elastic net regularization [28], (IV) L0-norm regularization [29], (V) a support vector classifier (SVC) [4], (VI) regularized discriminant function analysis (RDA) [30], (VII) a Gaussian process classifier (GPC) [31], and (VIII) Random forests (RF). A theoretical overview of these algorithms together with technical details on their implementation can be found in the S1 Appendix **(Description of learning algorithms)**.

### General procedure and cross validation scheme

**Fig 2:**
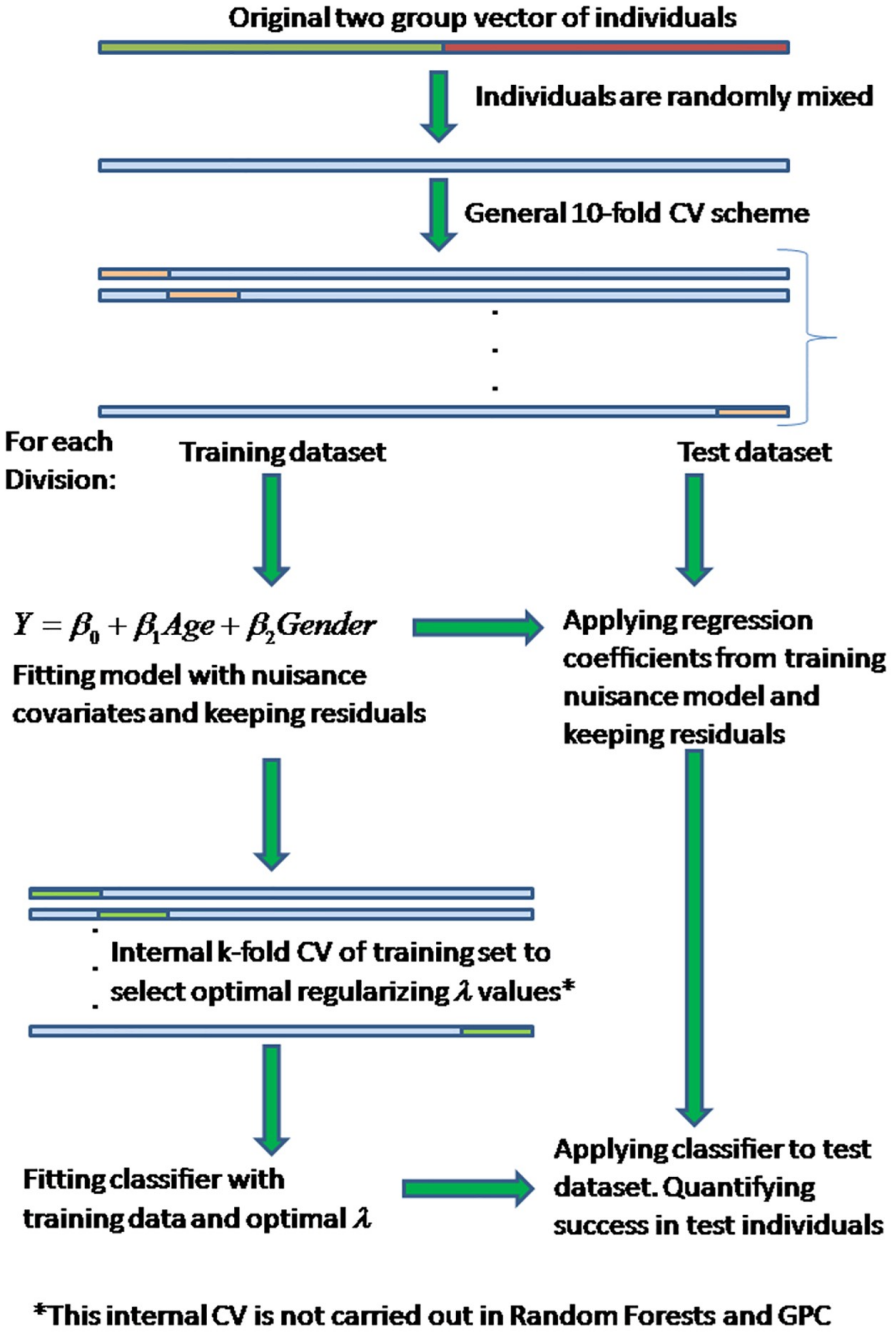
General cross validation scheme applied to evaluate the classification accuracy in all combinations of algorithms and data features. For most classifiers, cross-validation is used at two levels: at an outer level for training and testing and within each training sample to select the optimal values for the regularization parameters (delta). The effect of nuisance covariates on test data should be regressed out by using coefficients fitted in the training data. Individual performances are given as frequencies of test individuals successfully classified.

To have a non-biased assessment of the performance we applied the classifiers, which had been previously built on training samples, on a completely independent group of individuals (i.e. a test sample). A 10-fold cross validation scheme was followed to divide the original sample (made of all individuals belonging to the two groups) in 10 non-overlapping partitions [4]. For each partition individuals included were considered as the test sample, and the remaining individuals as the training sample. A graphical representation of the general procedure for evaluating classification accuracy is given in Fig 2.

For all classifiers but RF and GPC cross-validation is used at two levels: at an outer level the complete sample is divided in 10 parts for training and testing, but within each training sample a second internal cross-validation is usually carried out to select the optimal values for the regularization parameters. From a range of parameter values, those minimizing the classification error in this internal cross-validation are used to build de classifier, which later is applied to the test data to have an objective assessment of classification accuracy. This procedure is repeated 10 times (for each of the 10-fold partitions) generating 10 accuracy estimates.

To avoid over-optimistic results the effect of nuisance covariates on test data should be regressed out by using those coefficients fitted in the training data (i.e. test data should not be used in the fitting of nuisance covariates) (see Fig 2). Individual performances of each algorithm—feature combination are given as frequencies of test individuals successfully classified (assuming a *p(X)* > 0.5 threshold) and by other quantities such as the area under the (receiver operating) curve (AUC) [4]. The receiver operating curve (ROC), which is based on the relative performances considering the whole range of possible probability thresholds (from 0 to 1) has an area that ranges from 0.5 for classifiers without any prediction capability to 1 for perfectly classifying algorithms.

### Multi-class classifiers

Although many of the learning algorithms used here were initially designed for two group classifications, extensions have been built to deal with more than two groups simultaneously. Here we have applied three different approaches for simultaneous classification of the three groups. On the one hand, after performing the three possible pairwise classifications (among our three groups) we have assigned each test individual to the class with highest mean probability (i.e. a one-versus-one classification approach [4]). Alternatively, we have carried out classifications between each class and a merged class containing subjects from the two remaining classes, assigning test individuals to the non-merged class with highest probability (i.e. a one-versus-all approach [4]) and, finally, for those classifiers with inbuilt multiclass functionality (all but the L0-norm and SVC) we have used the methods available. These involved the regularized multinomial regression (for the ridge, lasso and elastic net), the multi-class regularized discriminant analysis, and the multi-class versions of the GPC and RF. It should be noted that in all three-class classifications we had a 0.333 probability of assigning, by chance, the individual to the correct class.

## Results

**Fig 3:**
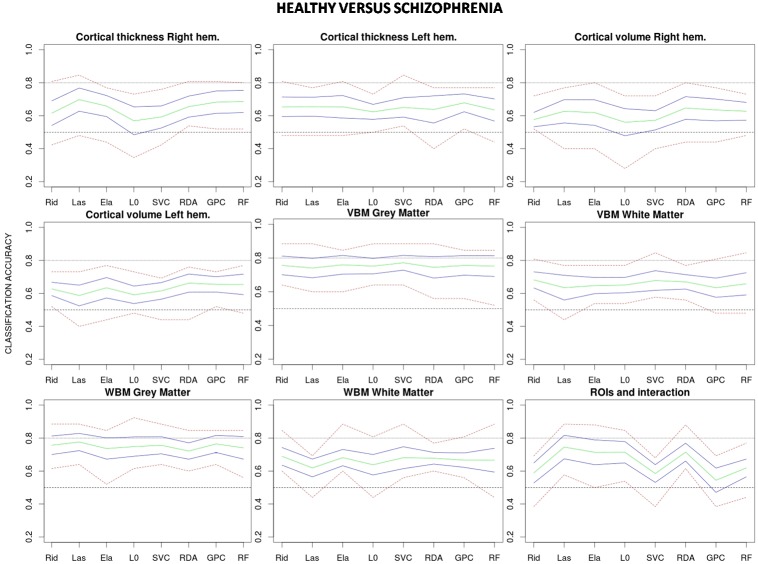
Classification accuracies for each combination of algorithm and feature type applied to the healthy vs. schizophrenia classification. Mean accuracy for the 10 test samples (in green), approximate 95% confidence interval for the mean accuracy (in blue) and highest and lowest accuracy values (in red) are shown for each combination. Rid: Ridge regression, Las: Lasso regression, Ela: Elastic net regularization, L0: L0-norm regularization, SVC: Support vector classifier, RDA: Regularized discriminant analysis, GPC: Gaussian process classifier, RF: Random forest.

**Fig 4:**
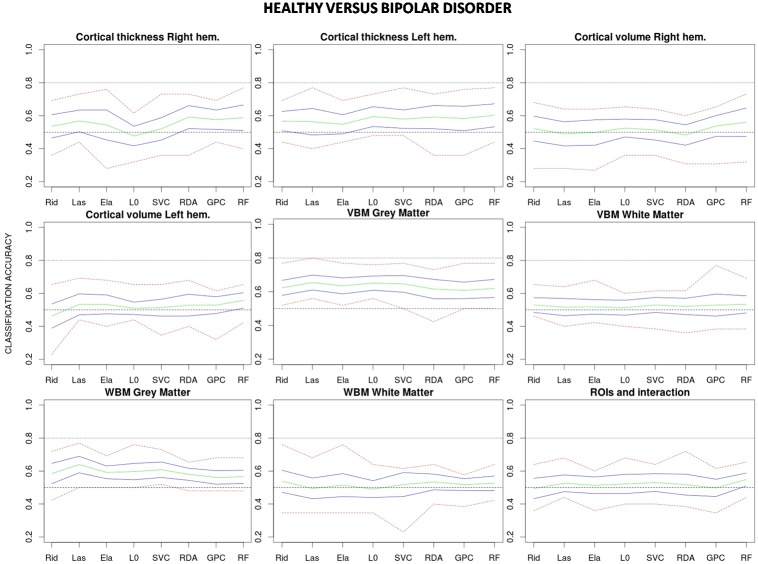
Classification accuracies for each combination of algorithm and feature type applied to the healthy vs. bipolar disorder classification. Mean accuracy for the 10 test samples (in green), approximate 95% confidence interval for the mean accuracy (in blue) and highest and lowest accuracy values (in red) are shown for each combination. Rid: Ridge regression, Las: Lasso regression, Ela: Elastic net regularization, L0: L0-norm regularization, SVC: Support vector classifier, RDA: Regularized discriminant analysis, GPC: Gaussian process classifier, RF: Random forest.

**Fig 5:**
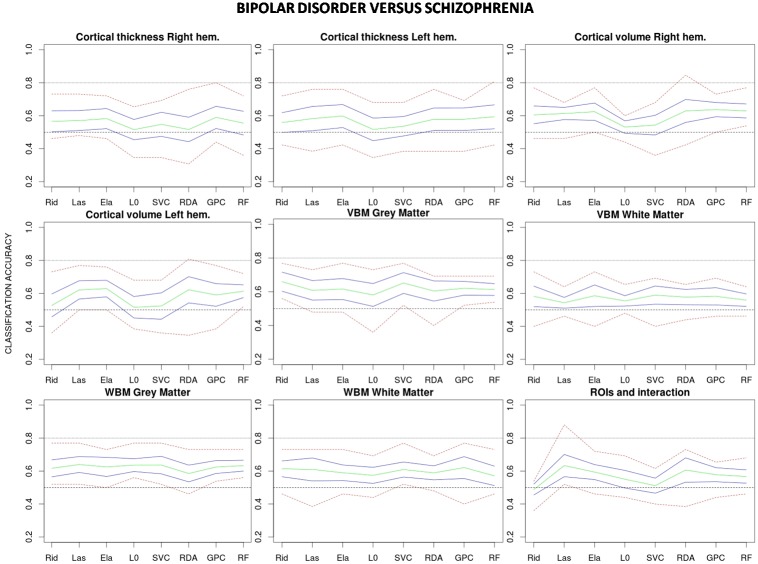
Classification accuracies for each combination of algorithm and feature type applied to the bipolar disorder vs. schizophrenia classification. Mean accuracy for the 10 test samples (in green), approximate 95% confidence interval for the mean accuracy (in blue) and highest and lowest accuracy values (in red) are shown for each combination. Rid: Ridge regression, Las: Lasso regression, Ela: Elastic net regularization, L0: L0-norm regularization, SVC: Support vector classifier, RDA: Regularized discriminant analysis, GPC: Gaussian process classifier, RF: Random forest.

**Fig 6:**
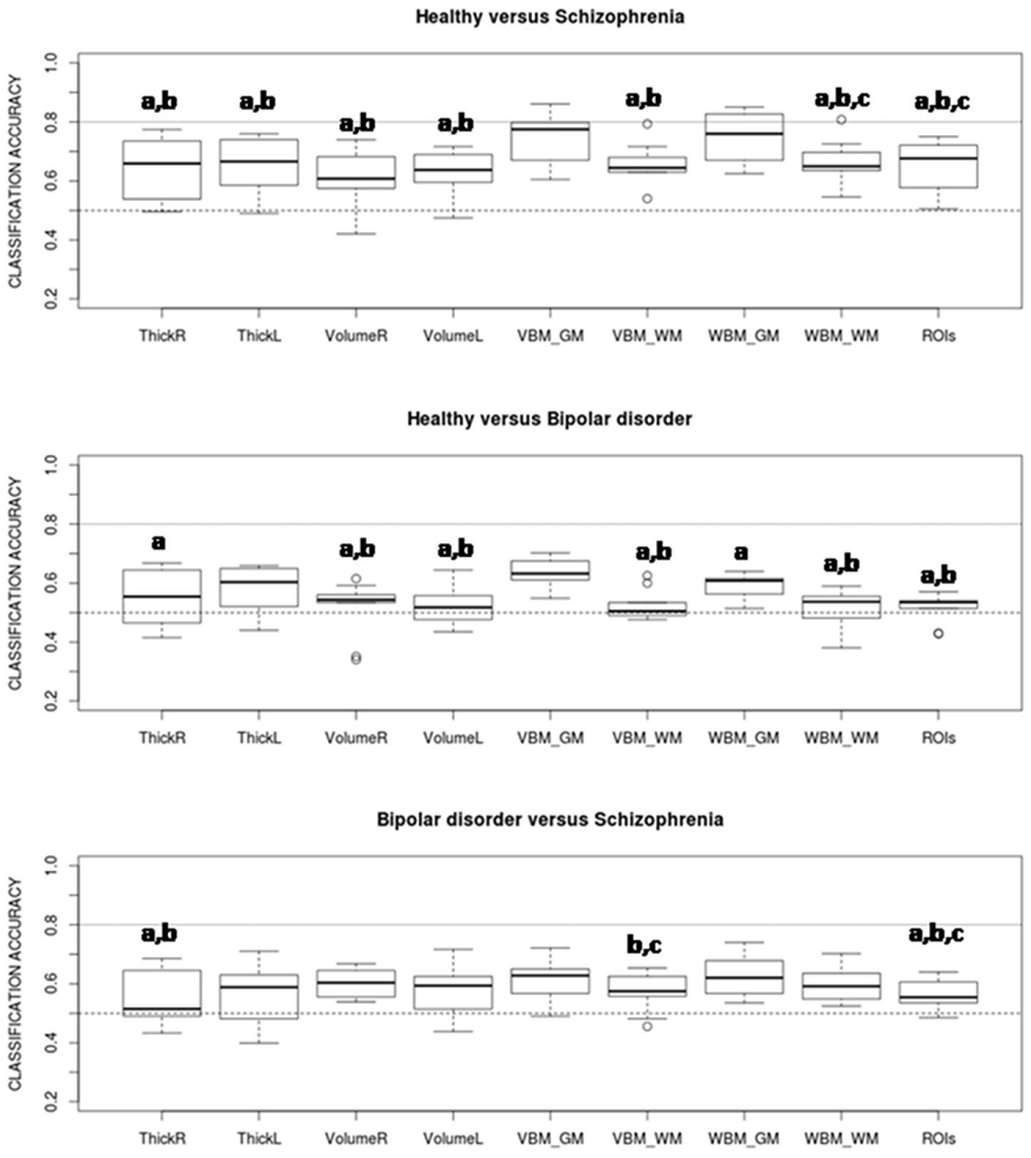
Accuracy rates averaged over all classifiers for the different feature types. Pairs of features showing significant differences from paired Wilcoxon tests (with p < 0.05) are signaled. Most of the significant differences involve a higher accuracy rate for grey matter VBM and WBM. a: significantly different from VBM_GM, b: significantly different from WBM_GM, c: significantly different from VolumeR.

**Fig 7:**
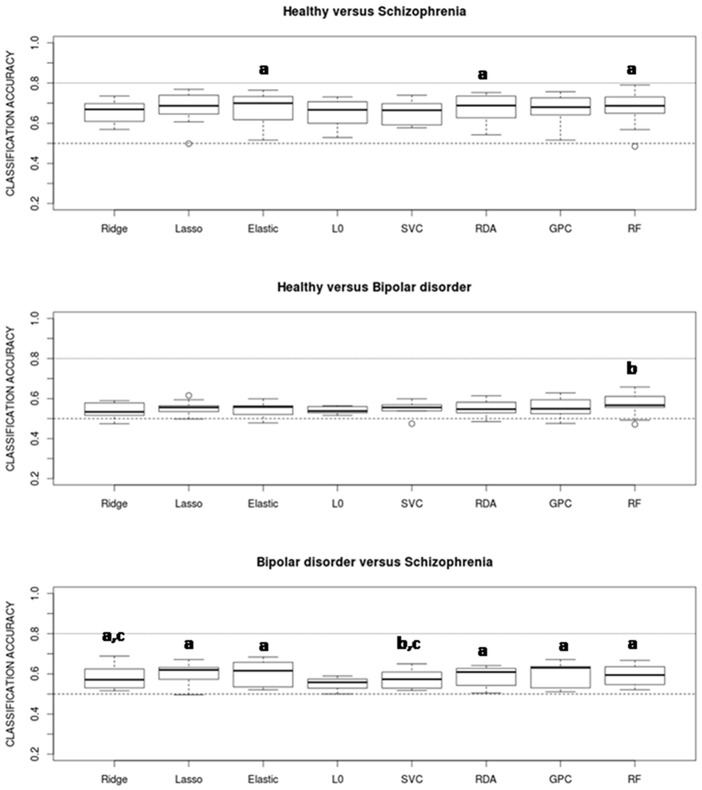
Accuracy rates averaged over all feature types for the eight classifiers. Pairs of classifiers showing significant differences from paired Wilcoxon tests (with p < 0.05) are marked. None of the classifiers clearly outperforms the others. a: significantly different from L0, b: significantly different from GPC, c: significantly different from Elastic.

Estimated classification accuracies for all possible combinations of algorithms and data features are shown in Fig 3 (healthy vs. schizophrenia), Fig 4 (healthy vs. bipolar) and Fig 5 (bipolar vs. schizophrenia). As a general trend, accuracies achieved in the healthy vs. schizophrenia classifications are higher than those observed in the healthy vs. bipolar and bipolar vs. schizophrenia classifications. On the other hand, although results vary depending on the algorithm and data feature, grey matter (GM) VBM (without data reduction) and WBM feature types show classification accuracies which equal or exceed those achieved by the other features. Indeed, when accuracy rates are averaged over classifiers GM-WBM, and in a higher degree GM-VBM, significantly outperform most of the other feature types in the healthy vs. schizophrenia and in the healthy vs. bipolar classifications (Fig 6). However, for the bipolar vs. schizophrenia classification this trend is less clear. In contrast, when classification rates are averaged over features and algorithms are compared, no single classifier outperforms the others (Fig 7), and a poor performance of the L0-norm classifier is the only distinctive and significant pattern. In Figs 6 and 7 the better performance of healthy vs. schizophrenia classifications is even more evident. Indeed, when Wilcoxon tests were run on the average-over-feature accuracies of Fig 7, accuracies were significantly higher for all algorithms in the healthy vs. schizophrenia pair (See S2 Appendix).

Classification levels achieved by the different algorithms when applied to grey matter VBM (the best performing feature type) are shown in Table 2. Best rates were attained in the healthy vs. schizophrenia classifications, with accuracies as high as 0.77 for the SVC, although no classifier significantly outperformed any other (Wilcoxon paired test at p < 0.05) (average accuracy over all classifiers equaled 0.75) while misclassification between healthy individuals and individuals with bipolar disorder was higher (averaged accuracy declined to 0.63) but again no single classifier outperformed any other classifier. Finally, the classification between both psychiatric disorders reported similar classification levels with a mean accuracy of 0.62, although here the ridge regression algorithm (with mean accuracy of 0.66) significantly outperformed the L0-norm classifier (mean accuracy of 0.58); Wilcoxon paired test p = 0.035. However, no other comparison was significant.

**Table 2:** Mean accuracy rate and area under the receiver operating curve (AUC) for the eight classifiers on VBM grey matter.

	Algorithm	Mean accuracy	5%limit	95%limit	AUC	5%limit	95%limit
Healthy vs. Schizophrenia	^a^ridge	0.756	0.701	0.806	0.836	0.788	0.885
	^b^lasso	0.741	0.683	0.792	0.82	0.769	0.872
	^c^elastic	0.76	0.703	0.812	0.815	0.762	0.869
	^d^L0 norm	0.752	0.712	0.795	0.835	0.785	0.884
	^e^SVC	0.772	0.731	0.812	0.85	0.804	0.896
	^f^RDA	0.745	0.683	0.804	---	---	---
	^g^GPC	0.756	0.699	0.805	0.828	0.778	0.878
	^h^RF	0.752	0.69	0.805	0.837	0.788	0.885
Healthy vs. Bipolar dis.	ridge	0.623	0.586	0.664	0.686	0.621	0.75
	lasso	0.655	0.616	0.7	0.702	0.639	0.766
	elastic	0.635	0.592	0.681	0.691	0.627	0.756
	L0 norm	0.651	0.613	0.69	0.706	0.643	0.769
	SVC	0.647	0.599	0.694	0.698	0.634	0.762
	RDA	0.616	0.557	0.668	---	---	---
	GPC	0.608	0.565	0.658	0.671	0.605	0.737
	RF	0.62	0.571	0.67	0.688	0.624	0.753
Bipolar dis. vs. Schizophrenia	ridge	0.66	0.605	0.716	0.692	0.627	0.756
	lasso	0.609	0.555	0.659	0.646	0.579	0.713
	elastic	0.616	0.562	0.676	0.689	0.624	0.753
	L0 norm	0.581	0.507	0.643	0.659	0.593	0.726
	SVC	0.652	0.593	0.712	0.696	0.632	0.761
	RDA	0.605	0.545	0.657	---	---	---
	GPC	0.621	0.583	0.661	0.696	0.632	0.76
	RF	0.613	0.581	0.646	0.685	0.621	0.75

Lower and upper limits for the 95% confidence intervals generated by bootstrap are also reported for these two quantities.

^a^ridge: Ridge regression,

^b^lasso: Lasso regression,

^c^elastic: Elastic net regularization,

^d^L0-norm: L0-norm regularization,

^e^SVC: Support vector classifier,

^f^RDA: Regularized discriminant analysis,

^g^GPC: Gaussian process classifier,

^h^RF: Random forest.

**Fig 8:**
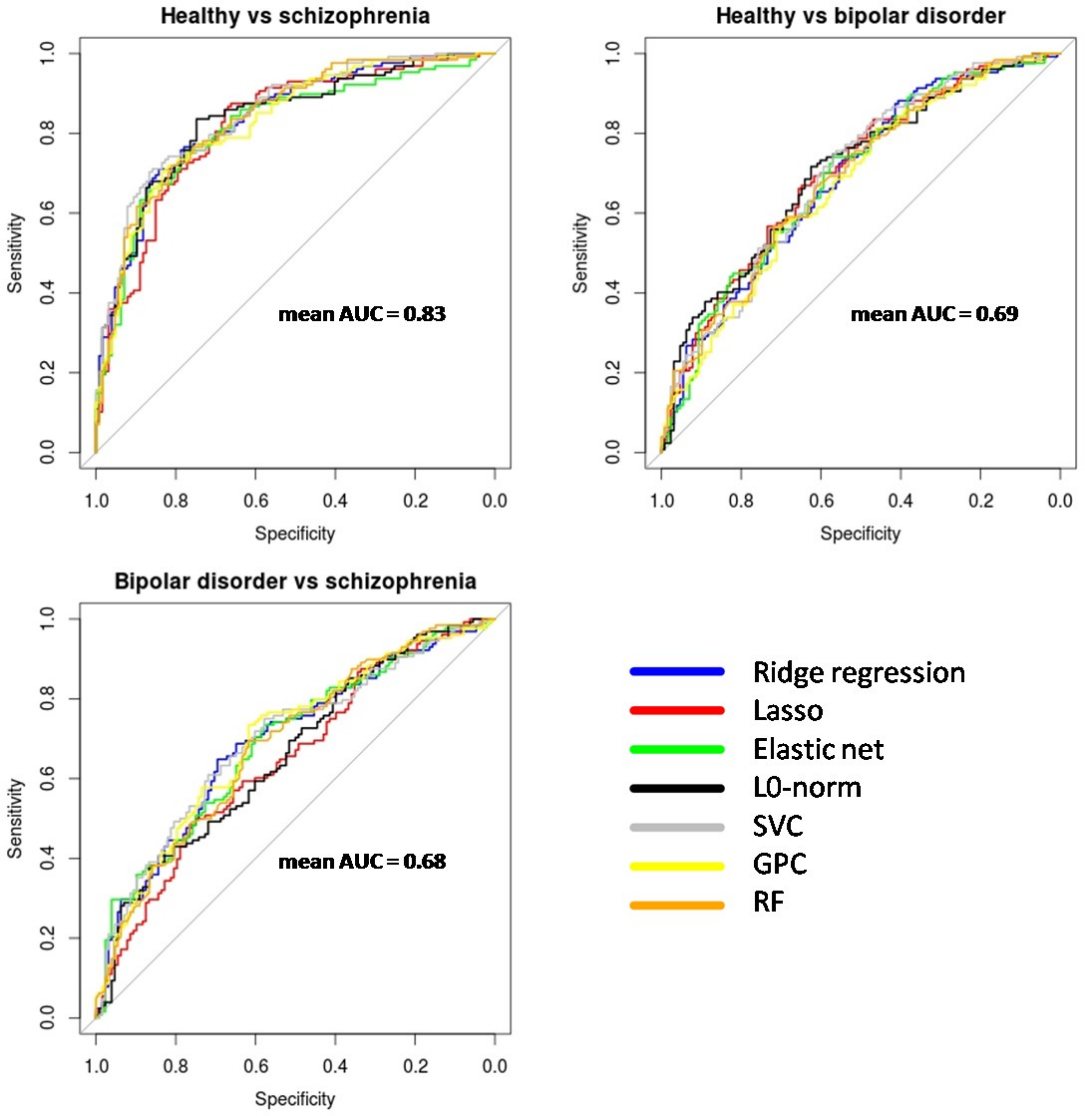
Receiver Operating Curves (ROCs) for the different classifiers applied to grey matter VBM. Best classification performances are observed in the healthy vs. schizophrenia classification. The overlap between curves in each plot points to similar classification levels attained by the different algorithms. AUC: Area under the receiver operating curve. There are no ROCs for the Regularized discriminant analysis because no reliable individual probabilities were available for this algorithm.

Fig 8 portrays receiver operating curves (ROC) for classifiers in the three different pairwise classifications (for all classifiers except the RDA, for which we did not have reliable estimates of individual probabilities). As expected by the similar classification rates previously reported, plotted curves had similar trajectories. Highest AUC levels were achieved in the healthy vs. schizophrenia classification with an average AUC of 0.83 (values for each classifier are given in Table 2) which declined to 0.69 for the healthy vs. bipolar disorder classification and to 0.68 in the classification between both disorders. A bootstrap based statistical test comparing the AUC between classifiers reported very few significant differences between algorithm performances. These only included a higher AUC for the SVC versus the elastic net regression (p = 0.005) and versus the GPC (p = 0.009) in the healthy vs. schizophrenia classification.

**Fig 9:**
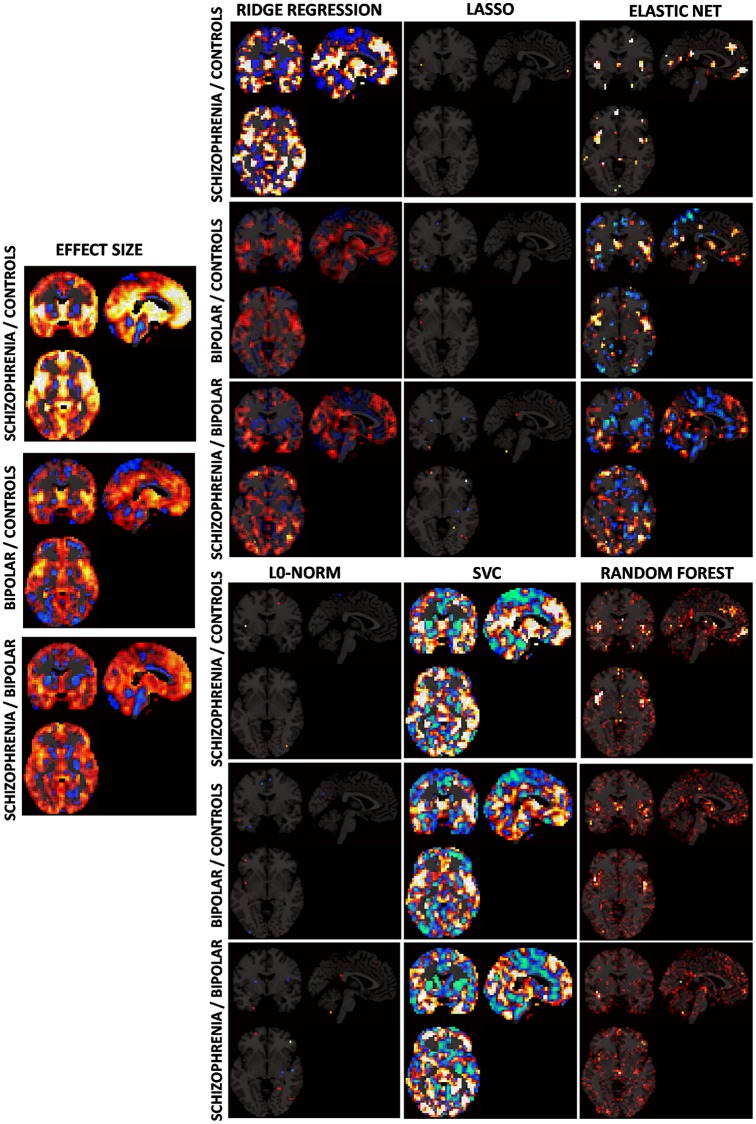
Brain maps of coefficients from fitted classifiers on grey matter VBM images together with effect size maps as given by standard univariate t-tests. Values for the random forest classifier are variable importance measures derived from the Gini index. Functions for the Gaussian process classifier and the regularized discriminant analysis did not provide fitted coefficients and maps were not available.

**Fig 10:**
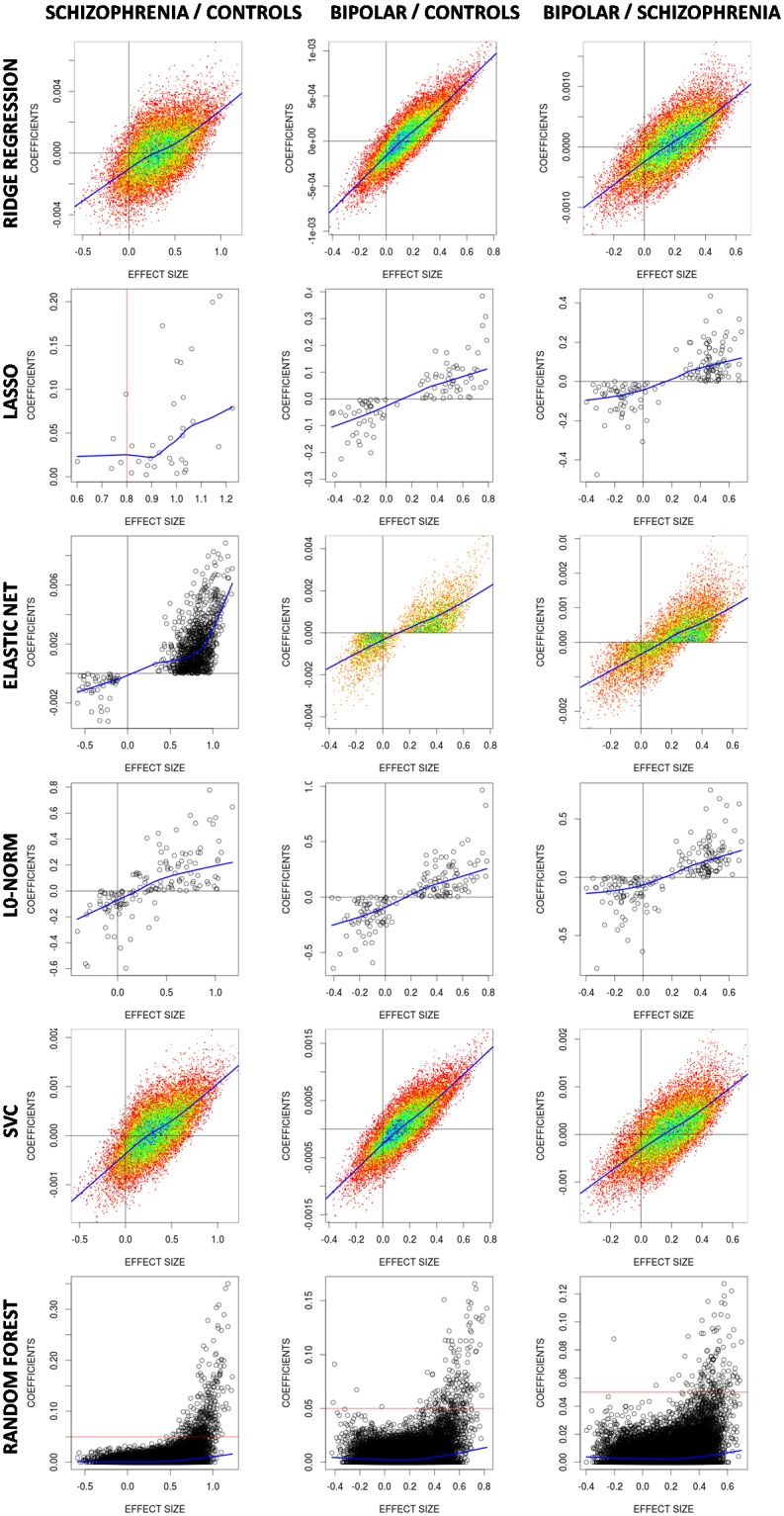
Plots of t-test based effect sizes (x-axis) versus (non-zero) coefficients from the different classifiers (y-axis) applied to grey matter VBM data. Non parametric local regression (lowess) lines are shown in blue. Random forest values are variable importances derived from the Gini index. No coefficients were available for the Gaussian process classifier and the regularized discriminant analysis.

To gain some insight on the inner functioning of algorithms applied to grey matter VBM data, maps of fitted coefficients and weights were obtained for most of the classifiers (see Fig 9). In Fig 9 coefficient maps are drawn together with maps of effect sizes, which were derived from standard univariate t-tests applied to each voxel (i.e. the standard method for generating maps of differences in group comparisons). Although the aspect of coefficient maps clearly differed among classifiers, there was a broad agreement between most prominent patterns and features in the effect size maps. A more quantitative view of such agreement is provided by plots of Fig 10 where, in most cases, a monotonic increasing relationship is shown between effect size and coefficient value. In those cases where this relationship was not clear (the lasso in controls vs. schizophrenia and all RF classifications) largest coefficients were still linked to voxels with largest effect sizes. Values of RF though, are not model coefficients but variable importance measures derived from the Gini index [4]. This agreement between coefficients and effect sizes links classifiers with likely anatomical group divergences.

**Fig 11:**
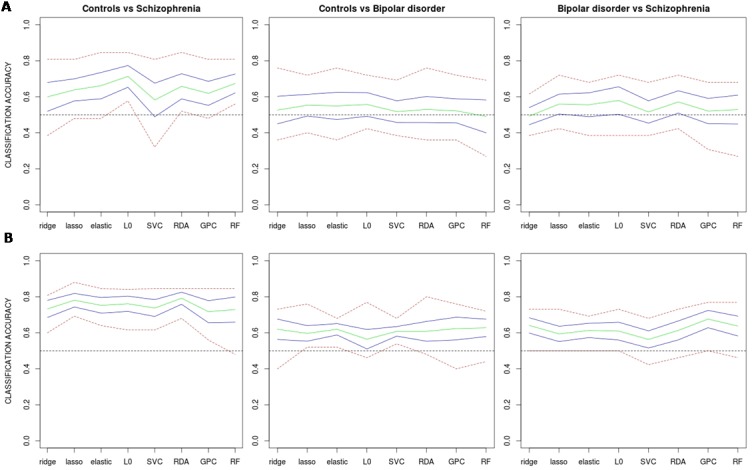
Estimated classification accuracies obtained by considering all feature types together as predictors. In (A) a principal component analysis was previously applied to the merged data to reduce computational burden and dimensionality, in (B) only the 1% of variables with largest t values as given by univariate two group comparisons was considered. Green line: mean accuracy for the 10 test samples; blue lines: approximate 95% confidence intervals for the mean accuracy; red line: highest and lowest accuracy values. ridge: Ridge regression, lasso: Lasso regression, elastic: Elastic net regularization, L0: L0-norm regularization, SVC: Support vector classifier, RDA: Regularized discriminant analysis, GPC: Gaussian process classifier, RF: Random forest.

When all information from the different data features was combined together, and after applying PCA for dimensionality reduction, classifiers reported accuracies clearly lower than those achieved only with grey matter VBM (without dimensionality reduction) (Fig 11A). And although most mean accuracies were higher than 0.5, bootstrap intervals revealed that for the two classifications involving bipolar subjects many of these were not significantly different from 0.5 (see Table 3). In contrast, when the top 1% of variables with largest t-values were considered, accuracies achieved levels very similar to those provided by grey matter VBM (see Fig 11B), and in all cases they were considered significantly larger than 0.5 (see bootstrap intervals in Table 3). In any case, however, performances were higher than those provided by grey matter VBM.

**Table 3:** Mean classification accuracies obtained by combining all data features together, after dimensionality reduction based on Principal Component Analysis (PCA Based combination), and after selecting only the 1% of variables with largest t values (t-thresholded combination).

	Algorithm	PCA based combination			t-thresholded combination		
		Mean accuracy	5%limit	95%limit	Mean accuracy	5%limit	95%limit
Healthy vs. schizophrenia	^a^ridge	0.6	0.516	0.673	0.733	0.686	0.776
	^b^lasso	0.638	0.584	0.698	0.781	0.747	0.816
	^c^elastic	0.662	0.598	0.728	0.752	0.711	0.791
	^d^L0 norm	0.713	0.655	0.771	0.761	0.717	0.798
	^e^SVC	0.582	0.495	0.675	0.737	0.693	0.781
	^f^RDA	0.658	0.593	0.725	0.792	0.758	0.823
	^g^GPC	0.619	0.561	0.688	0.717	0.657	0.776
	^h^RF	0.674	0.624	0.725	0.729	0.657	0.792
Healthy vs. bipolar dis.	ridge	0.526	0.455	0.598	0.619	0.562	0.662
	lasso	0.553	0.5	0.612	0.596	0.561	0.639
	elastic	0.549	0.484	0.626	0.619	0.586	0.646
	L0 norm	0.557	0.495	0.62	0.564	0.518	0.618
	SVC	0.518	0.461	0.574	0.608	0.583	0.632
	RDA	0.529	0.464	0.603	0.608	0.563	0.663
	GPC	0.522	0.459	0.585	0.623	0.561	0.677
	RF	0.491	0.405	0.576	0.627	0.575	0.67
Bipolar dis. vs. schizophrenia	ridge	0.492	0.446	0.537	0.641	0.601	0.678
	lasso	0.56	0.506	0.614	0.594	0.557	0.637
	elastic	0.556	0.493	0.614	0.613	0.572	0.651
	L0 norm	0.579	0.507	0.65	0.609	0.564	0.657
	SVC	0.516	0.456	0.573	0.563	0.518	0.607
	RDA	0.571	0.513	0.628	0.613	0.564	0.661
	GPC	0.521	0.457	0.586	0.676	0.627	0.718
	RF	0.529	0.453	0.599	0.637	0.583	0.686

Limits for 95% confidence intervals are based on bootstrap.

^a^ridge: Ridge regression,

^b^lasso: Lasso regression,

^c^elastic: Elastic net regularization,

^d^L0-norm: L0-norm regularization,

^e^SVC: Support vector classifier,

^f^RDA: Regularized discriminant analysis,

^g^GPC: Gaussian process classifier,

^h^RF: Random forest.

**Fig 12:**
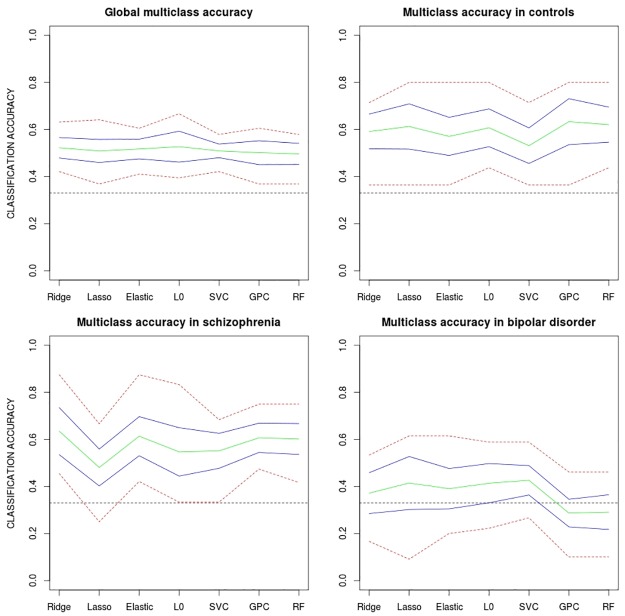
Classification accuracies generated by multi-class classifiers on grey matter VBM using the one-vs-one approach. All algorithms were used (except the regularized discriminant function analysis, which did not report reliable class probabilites). Overall accuracies are plotted together with accuracies for the three groups separately. Green line: mean accuracy for the 10 test samples; blue lines: approximate 95% confidence intervals for the mean accuracy; red line: highest and lowest accuracy values. ridge: Ridge regression, lasso: Lasso regression, elastic: Elastic net regularization, L0-norm: L0-norm regularization, SVC: Support vector classifier, GPC: Gaussian process classifier, RF: Random forest.

Accuracies from one-versus-one multi-class classifications on grey matter VBM were, in general, lower than those delivered by pairwise classifications (see Fig 12). Furthermore, although significant predictive power was still found for controls (with an average accuracy of 60%) and for schizophrenia (with an average accuracy of 57%) classification rates for bipolar patients (with an average accuracy of 37%) were quite close to the 33% expected by chance. Indeed, most classifiers included the 33% inside the bootstrap confidence intervals (see Table 4) suggesting that multi-class algorithms do not classify bipolar patients reliably. Results from the other two multi-class schemes (one-versus-all and inbuilt multiclass) delivered similar levels of accuracy than those of the one-versus-one design (see Tables 4 and 5). While mean overall accuracy was 51% for the one-versus-one approach a value of 52% was attained for both one-versus-all and inbuilt approaches. Again, all classifiers showed a significant predictive power for the control group (average of 64% for both schemes) and schizophrenia group (63% and 60%) but no reliable prediction power was found for the bipolar group (average accuracy of 30% and 32% respectively).

**Table 4:** Mean accuracies obtained by all classifiers (apart from the regularized discriminant function analysis) using a one-vs-one and a one-vs-all multi-class approach on grey matter VBM images. Lower and upper limits for the 95% bootstrap confidence intervals are also reported. 0.333 is the expected accuracy when no real predictive power is present.

	Algorithm	One-versus-one			One-versus-all		
		Mean accuracy	5%limit	95%limit	Mean accuracy	5%limit	95%limit
Overall	^a^ridge	0.522	0.479	0.562	0.514	0.475	0.551
	^b^lasso	0.509	0.462	0.554	0.527	0.483	0.566
	^c^elastic	0.517	0.478	0.557	0.527	0.493	0.559
	^d^L0 norm	0.527	0.464	0.588	0.566	0.525	0.602
	^e^SVC	0.509	0.481	0.537	0.491	0.443	0.538
	^f^GPC	0.501	0.452	0.547	0.515	0.463	0.563
	^g^RF	0.496	0.452	0.538	0.483	0.436	0.525
Controls	ridge	0.592	0.512	0.657	0.632	0.562	0.694
	lasso	0.613	0.514	0.698	0.584	0.507	0.656
	elastic	0.571	0.494	0.647	0.639	0.558	0.707
	L0 norm	0.607	0.535	0.688	0.632	0.56	0.709
	SVC	0.531	0.463	0.608	0.66	0.537	0.772
	GPC	0.633	0.543	0.72	0.656	0.564	0.741
	RF	0.621	0.548	0.688	0.68	0.591	0.764
Schizophrenia	ridge	0.635	0.544	0.738	0.631	0.544	0.74
	lasso	0.481	0.409	0.556	0.588	0.524	0.654
	elastic	0.614	0.542	0.694	0.621	0.545	0.704
	L0 norm	0.547	0.451	0.648	0.636	0.555	0.715
	SVC	0.551	0.48	0.617	0.629	0.531	0.723
	GPC	0.607	0.55	0.668	0.66	0.589	0.735
	RF	0.602	0.539	0.666	0.631	0.564	0.696
Bipolar dis.	ridge	0.372	0.285	0.448	0.282	0.185	0.363
	lasso	0.415	0.301	0.516	0.423	0.316	0.516
	elastic	0.391	0.315	0.475	0.319	0.254	0.387
	L0 norm	0.414	0.336	0.492	0.428	0.372	0.492
	SVC	0.426	0.369	0.487	0.204	0.128	0.286
	GPC	0.287	0.236	0.344	0.255	0.209	0.303
	RF	0.291	0.218	0.367	0.164	0.103	0.221

^a^ridge: Ridge regression,

^b^lasso: Lasso regression,

^c^elastic: Elastic net regularization,

^d^L0-norm: L0-norm regularization,

^e^SVC: Support vector classifier,

^f^GPC: Gaussian process classifier,

^g^RF: Random forest.

**Table 5:** Mean accuracies obtained by classifiers that provide inbuilt multiclass functionality (all but the L0-norm and the support vector classifiers). Lower and upper limits for the 95% bootstrap confidence intervals are also reported. 0.333 is the expected accuracy when no real predictive power is present.

	Algorithm	Mean accuracy	5%limit	95%limit
Overall	^a^ridge	0.533	0.48	0.58
	^b^lasso	0.54	0.511	0.576
	^c^elastic	0.538	0.506	0.575
	^d^RDA	0.475	0.434	0.517
	^e^GPC	0.504	0.456	0.551
	^f^RF	0.507	0.458	0.557
Controls	ridge	0.612	0.535	0.683
	lasso	0.662	0.559	0.753
	elastic	0.65	0.563	0.749
	RDA	0.606	0.515	0.698
	GPC	0.643	0.55	0.731
	RF	0.646	0.568	0.719
Schizophrenia	ridge	0.629	0.542	0.718
	lasso	0.623	0.522	0.743
	elastic	0.627	0.585	0.676
	RDA	0.555	0.471	0.647
	GPC	0.619	0.553	0.692
	RF	0.602	0.538	0.67
Bipolar dis.	ridge	0.358	0.274	0.449
	lasso	0.359	0.305	0.417
	elastic	0.34	0.286	0.384
	RDA	0.287	0.2	0.368
	GPC	0.279	0.224	0.324
	RF	0.306	0.239	0.379

^a^ridge: Ridge regression,

^b^lasso: Lasso regression,

^c^elastic: Elastic net regularization,

^d^RDA: Regularized discriminant function analysis,

^e^GPC: Gaussian process classifier,

^f^RF: Random forest.

## Discussion and conclusions

After applying the eight classifiers on the different feature types we can outline some general conclusions. First, it seems that while the election of the feature type may be of relevance to achieve an optimal classification, the choice of classifier is not important. Most classifiers provide similar levels of accuracy when an adequate feature type is selected. Specifically, for the three pairwise classifications carried out here with patients with psychosis, grey matter VBM and, to a minor extent, grey matter WBM are the feature types leading to highest accuracies. For them no single classifier clearly outperforms the others. This is rather surprising since, although it has been recently proven that some of the applied classifiers have clear mathematical similarities [4] it is also clear that some of them are unmistakably different. The most obvious case being the Random forest classifier, which binarizes continuous variables by partitioning the feature space, and is not constrained by the additivity found in logistic regressions and support vector classifiers. This same result, though, has been previously reported by Khondoker et al. [32] whom, in a classification involving patients with Alzheimer and controls, showed that as effect size (i.e. the real discriminative power of data) increased different classifiers tended to achieve similar levels of classification accuracy, making the choice of algorithm less relevant. A distribution of observations in the multidimensional feature space largely following an unstructured pattern could be a plausible explanation for our results. Such a distribution with unstructured noise would not be better classified by any complex function than a hyperplane, which is a geometrical feature that all classifiers are to a large extent able to generate, and this would eventually lead to similar classification accuracies. We have also seen in Fig 9 that, in spite of working differently, classifiers give largest weights to voxels located in the same or similar areas, extracting and using similar information from the VBM images. As well, there are reasonable explanations for the best classifying performance of grey matter VBM. First, while substantial white matter abnormalities have been described in both schizophrenic and bipolar patients through diffusion MRI [33, 34], such patterns have not been as clear in the few VBM studies analyzing white matter, at least in schizophrenia [35]. On the other hand, lower accuracies delivered by region of interest measures are attributable to the intrinsic loss of information caused by spatial averaging of high resolution data. Finally, the poorer performance of both vertex based cortical features may be related to their restricted spatial extent, which excludes all subcortical structures. In addition, major structural abnormalities in schizophrenia and bipolar disorder have been described in the medial frontal cortex and the insulas [35, 36] which are regions with high topological complexity.

It should also be noted that the primary objective of this study was the comparison of commonly used classifiers and feature types for classification in psychosis, intentionally leaving many other existing classifiers, subtypes and variants untested. Neither it was of interest to attain particularly high accuracies. Indeed, when performances in our study are compared to those found in other schizophrenia vs. healthy classifications reported in the recent revision by Wolfers et al. [7], they are average. The mean accuracy rate found for the bipolar vs. control classification based on the VBM data (63%) is also similar to values reported by the few studies analyzing the same classification on sMRI: 60% [14], 66% [15], 73% [16] with the exception of Bansal et al. [13] that achieved a surprisingly high classification accuracy of 98%. Finally, the only study directly classifying bipolar vs. schizophrenic patients [14] reported a classification accuracy of 88%, which is clearly higher than ours (62%). However, when they applied the fitted classifiers to an external sample, their classification accuracy descended to 65%.

Yet, most previous studies used smaller sample sizes, sometimes significantly smaller than ours, making their results less reliable. In our study, we have used large and well balanced samples and we have paid special attention in keeping the independence between test and training sets throughout all the image processing steps in order to avoid unintended biases and overoptimistic classification estimates. Also, when running the different classifiers we have noticed the relevance of carefully choosing the range of possible parameter values in the training phase which, if ignored, would lead to clearly suboptimal classification rates. Since our training samples had (nearly) equal number of individuals, classification rates assumed equal prior probabilities (of 0.5) for all classes. In real situations, though, this equality will sometimes not be met, and when using other priors, accuracy rates will be different from those reported here.

Similarities observed between effect sizes and classifier coefficients relate the later with apparent anatomical divergences, bringing some insight on the way classifiers use information from the images. However, such relation will hold true only if effect sizes contain patterns of real abnormality. Indeed, for both group pairs involving controls and patients we have found the highest effect sizes in areas, like the insulas and the medial frontal cortex, which have consistently reported as having grey matter reductions in VBM meta-analyses of both psychotic disorders [35, 36]. Still, a close agreement between effect sizes and fitted coefficients should not be expected as the former simply report univariate between group dissimilarities while the later are weights from multivariate predictive models that, in some cases (e.g. Random Forests) have a very complex nature. Also, in settings with many more features than cases and with high levels of spatial autocorrelation (as it occurs in sMRI images), sparse classifiers like the Lasso or the L0-norm may lead to an extremely large number of competing models having optimized prediction capabilities [4].

The decrease in classification accuracies observed when using combined features and PCA reduction was unexpected. In contrast to results in [25], merging information from different feature types did not bring any improvement. But unlike Wang et al. [25] which combined different MRI modalities (sMRI and resting state functional MRI), we have derived all features from the same T1 images (expecting higher levels of redundancy between data features). Furthermore, dimensionality reduction through principal components does not seem to have retained the most relevant information, as grey mater VBM clearly provided better classifying accuracies. In contrast, feature selection based on the t statistic has clearly been more successful in retaining the relevant information from the combined features, although grey mater VBM classification rates have not been surpassed by this approach.

Reductions found in multi-class classifications are easily explained by the presence of a third competing class in each classification. Here, the feature space should be divided in three excluding areas by the algorithm, thus increasing the probability of misclassification. Such effect is particularly noticeable in the bipolar disorder group, where classification levels do not depart significantly from what would be expected by chance (33%). This is likely due to the fact that, as made evident by the effect size maps of Fig 9, VBM intensities in bipolar patients tend to be located between those observed in controls and in patients with schizophrenia (i.e. patterns of abnormality in bipolar disorder are similar to those in schizophrenia but less intense). Such intermediate position between two competing classes has probably led to the higher misclassification rates observed in this clinical group. This result seems to be quite consistent as it has been replicated by the three multi-class schemes applied (the one-vs-one, the one-vs-all and the inbuilt multi-class approach) which have delivered similar correct classification rates. In any case, the lower accuracies observed in bipolar patients have practical implications for sMRI based classification in psychosis. Further lines of research include the optimal combination of the different classifiers to increase the currently reported accuracies. The inclusion of data features derived from other MRI modalities such as functional connectivity maps or diffusion based measures including fractional anisotropy and mean diffusivity may also allow achieving higher classification accuracies.

Finally, as an added feature of this study we also provide MRIPredict, a free tool for SPM, FSL and R that allows an easy specification of the MRI datasets, of confounds and covariates, of cross-validation parameters and of voxelwise models to be fit (this software is available at https://www.nitrc.org/projects/mripredict/). MRIPredict applies regularized logistic regression from the Glmnet library [37] and saves the models in MNI space, thus allowing a later application to new scans from other sites, even if they have different voxel dimensions. It must be noted, then, that the accuracy of the new predictions may be limited if the new scans show important methodological differences with the scans used to fit the model.

In summary, from our exhaustive analysis of algorithms and data features we conclude that while grey matter VBM is the feature of choice for sMRI based classification in psychosis, the selection of classifier is not relevant (most have similar performance levels). We also conclude that the combination of different features types (derived from the same T1 images) do not seem to increase classification accuracies over classification rates achieved by grey matter VBM. Finally, multi-class classifications considering the three groups simultaneously have made evident a lack of predictive power for the bipolar group. This is probably due to its intermediate anatomical features, located between those observed in healthy controls and those found in patients with schizophrenia. We provide a new software tool that we hope will help many researchers conduct optimized voxelwise predictions.

## Supporting information

S1 Appendix Description of learning algorithms. (DOC)

S2 Appendix Results from comparing average-over-feature accuracies in the three group pairs. (DOC)

## References

[1] Deo RC. Machine Learning in Medicine. Circulation. 2015;132(20):1920–30. 10.1161/CIRCULATIONAHA.115.001593 26572668 PMC5831252

[2] Wang S, Summers RM. Machine learning and radiology. Medical image analysis. 2012;16(5):933–51. 10.1016/j.media.2012.02.005 22465077 PMC3372692

[3] Pereira F, Mitchell T, Botvinick M. Machine learning classifiers and fMRI: a tutorial overview. NeuroImage. 2009;45(1 Suppl):S199–209. 10.1016/j.neuroimage.2008.11.007 19070668 PMC2892746

[4] Hastie T, Tibshirani R, Friedman JK. The elements of statistical learning Data mining, inference and prediction. second ed New York: Springer; 2009.

[5] Falahati F, Westman E, Simmons A. Multivariate data analysis and machine learning in Alzheimer's disease with a focus on structural magnetic resonance imaging. Journal of Alzheimer's disease: JAD. 2014;41(3):685–708. 10.3233/JAD-131928 24718104

[6] Patel MJ, Khalaf A, Aizenstein HJ. Studying depression using imaging and machine learning methods. NeuroImage Clinical. 2016;10:115–23. 10.1016/j.nicl.2015.11.003 26759786 PMC4683422

[7] Wolfers T, Buitelaar JK, Beckmann CF, Franke B, Marquand AF. From estimating activation locality to predicting disorder: A review of pattern recognition for neuroimaging-based psychiatric diagnostics. Neuroscience and biobehavioral reviews. 2015;57:328–49. 10.1016/j.neubiorev.2015.08.001 26254595

[8] Zacharaki EI, Wang S, Chawla S, Soo Yoo D, Wolf R, Melhem ER, et al Classification of brain tumor type and grade using MRI texture and shape in a machine learning scheme. Magnetic resonance in medicine: official journal of the Society of Magnetic Resonance in Medicine / Society of Magnetic Resonance in Medicine. 2009;62(6):1609–18. 10.1002/mrm.22147 PMC2863141 19859947

[9] Sweeney EM, Vogelstein JT, Cuzzocreo JL, Calabresi PA, Reich DS, Crainiceanu CM, et al A comparison of supervised machine learning algorithms and feature vectors for MS lesion segmentation using multimodal structural MRI. PloS one. 2014;9(4):e95753 10.1371/journal.pone.0095753 24781953 PMC4004572

[10] Robinson ME, O'Shea AM, Craggs JG, Price DD, Letzen JE, Staud R. Comparison of machine classification algorithms for fibromyalgia: neuroimages versus self-report. The journal of pain: official journal of the American Pain Society. 2015;16(5):472–7. 25704840 10.1016/j.jpain.2015.02.002 PMC4424119

[11] Aguilar C, Westman E, Muehlboeck JS, Mecocci P, Vellas B, Tsolaki M, et al Different multivariate techniques for automated classification of MRI data in Alzheimer's disease and mild cognitive impairment. Psychiatry research. 2013;212(2):89–98. 10.1016/j.pscychresns.2012.11.005 23541334

[12] Casanova R, Hsu FC, Espeland MA, Alzheimer's Disease Neuroimaging I. Classification of structural MRI images in Alzheimer's disease from the perspective of ill-posed problems. PloS one. 2012;7(10):e44877 10.1371/journal.pone.0044877 23071501 PMC3468621

[13] Bansal R, Staib LH, Laine AF, Hao X, Xu D, Liu J, et al Anatomical brain images alone can accurately diagnose chronic neuropsychiatric illnesses. PloS one. 2012;7(12):e50698 10.1371/journal.pone.0050698 23236384 PMC3517530

[14] Schnack HG, Nieuwenhuis M, van Haren NE, Abramovic L, Scheewe TW, Brouwer RM, et al Can structural MRI aid in clinical classification? A machine learning study in two independent samples of patients with schizophrenia, bipolar disorder and healthy subjects. NeuroImage. 2014;84:299–306. 10.1016/j.neuroimage.2013.08.053 24004694

[15] Serpa MH, Ou Y, Schaufelberger MS, Doshi J, Ferreira LK, Machado-Vieira R, et al Neuroanatomical classification in a population-based sample of psychotic major depression and bipolar I disorder with 1 year of diagnostic stability. BioMed research international. 2014;2014:706157 10.1155/2014/706157 24575411 PMC3915628

[16] Rocha-Rego V, Jogia J, Marquand AF, Mourao-Miranda J, Simmons A, Frangou S. Examination of the predictive value of structural magnetic resonance scans in bipolar disorder: a pattern classification approach. Psychological medicine. 2014;44(3):519–32. 10.1017/S0033291713001013 23734914 PMC3880067

[17] Del Ser T, Gonzalez-Montalvo JI, Martinez-Espinosa S, Delgado-Villapalos C, Bermejo F. Estimation of premorbid intelligence in Spanish people with the Word Accentuation Test and its application to the diagnosis of dementia. Brain and cognition. 1997;33(3):343–56. 10.1006/brcg.1997.0877 9126399

[18] Fischl B, van der Kouwe A, Destrieux C, Halgren E, Segonne F, Salat DH, et al Automatically parcellating the human cerebral cortex. Cerebral cortex. 2004;14(1):11–22. 14654453 10.1093/cercor/bhg087

[19] Lerch JP, Evans AC. Cortical thickness analysis examined through power analysis and a population simulation. NeuroImage. 2005;24(1):163–73. 10.1016/j.neuroimage.2004.07.045 15588607

[20] Ashburner J. Computational anatomy with the SPM software. Magnetic resonance imaging. 2009;27(8):1163–74. 10.1016/j.mri.2009.01.006 19249168

[21] Smith SM. Fast robust automated brain extraction. Human brain mapping. 2002;17(3):143–55. 10.1002/hbm.10062 12391568 PMC6871816

[22] Jenkinson M, Beckmann CF, Behrens TE, Woolrich MW, Smith SM. Fsl. NeuroImage. 2012;62(2):782–90. 10.1016/j.neuroimage.2011.09.015 21979382

[23] Canales-Rodriguez EJ, Radua J, Pomarol-Clotet E, Sarro S, Aleman-Gomez Y, Iturria-Medina Y, et al Statistical analysis of brain tissue images in the wavelet domain: wavelet-based morphometry. NeuroImage. 2013;72:214–26. 10.1016/j.neuroimage.2013.01.058 23384522

[24] Desikan RS, Segonne F, Fischl B, Quinn BT, Dickerson BC, Blacker D, et al An automated labeling system for subdividing the human cerebral cortex on MRI scans into gyral based regions of interest. NeuroImage. 2006;31(3):968–80. 10.1016/j.neuroimage.2006.01.021 16530430

[25] Wang L, Shen H, Tang F, Zang Y, Hu D. Combined structural and resting-state functional MRI analysis of sexual dimorphism in the young adult human brain: an MVPA approach. NeuroImage. 2012;61(4):931–40. 10.1016/j.neuroimage.2012.03.080 22498657

[26] Dai Z, Yan C, Wang Z, Wang J, Xia M, Li K, et al Discriminative analysis of early Alzheimer's disease using multi-modal imaging and multi-level characterization with multi-classifier (M3). NeuroImage. 2012;59(3):2187–95. 10.1016/j.neuroimage.2011.10.003 22008370

[27] Tibshirani R. Regression Shrinkage and Selection via the lasso. Journal of the Royal Statistical Society Series B. 1996;58:267–88.

[28] Zou H, Hastie T. Regularization and Variable Selection via the Elastic Net. Journal of the Royal Statistical Society, Series B. 2005;67:301–20.

[29] Bühlmann P, Meier L, van de Geer S. Discussion: A significance test for the lasso. The Annals of Statistics. 2014;42:469–77. 10.1214/13-AOS1175 PMC4285373 25574062

[30] Guo Y, Hastie T, Tibshirani R. Regularized discriminant analysis and its application in microarrays. Biostatistics. 2005;1:1_18. 10.1093/biostatistics/kxj035 16603682

[31] Matthias S. Gaussian Processes for Machine Learning. International Journal of Neural Systems. 2004;14:69–104. 10.1142/S0129065704001899 15112367

[32] Khondoker M, Dobson R, Skirrow C, Simmons A, Stahl D. A comparison of machine learning methods for classification using simulation with multiple real data examples from mental health studies. Statistical methods in medical research. 2013. 10.1177/0962280213502437 PMC5081132 24047600

[33] Nortje G, Stein DJ, Radua J, Mataix-Cols D, Horn N. Systematic review and voxel-based meta-analysis of diffusion tensor imaging studies in bipolar disorder. Journal of affective disorders. 2013;150(2):192–200. 10.1016/j.jad.2013.05.034 23810479

[34] Pomarol-Clotet E, Canales-Rodriguez EJ, Salvador R, Sarro S, Gomar JJ, Vila F, et al Medial prefrontal cortex pathology in schizophrenia as revealed by convergent findings from multimodal imaging. Molecular psychiatry. 2010;15(8):823–30. 10.1038/mp.2009.146 20065955 PMC2927029

[35] Bora E, Fornito A, Radua J, Walterfang M, Seal M, Wood SJ, et al Neuroanatomical abnormalities in schizophrenia: a multimodal voxelwise meta-analysis and meta-regression analysis. Schizophrenia research. 2011;127(1–3):46–57. 10.1016/j.schres.2010.12.020 21300524

[36] Ellison-Wright I, Bullmore E. Anatomy of bipolar disorder and schizophrenia: a meta-analysis. Schizophrenia research. 2010;117(1):1–12. 10.1016/j.schres.2009.12.022 20071149

[37] Friedman J, Hastie T, Tibshirani R. Regularization Paths for Generalized Linear Models via Coordinate Descent. Journal of statistical software. 2010;33(1):1–22. 20808728 PMC2929880

