# Supplementary material for: Evaluation of machine learning algorithms and structural features for optimal MRI-based diagnostic prediction in psychosis
Source: PLoS One. 2017 Apr 20;12(4):e0175683. doi: 10.1371/journal.pone.0175683 (PMC5398548; doi:10.1371/journal.pone.0175683)
Supplement: S1 Appendix — (DOC) [file pone.0175683.s001.doc]

**S1 Appendix. Description of learning algorithms**

In this appendix we provide a theoretical overview for the eight algorithms evaluated, together with technical details on their implementation.

I) **Ridge logistic regression**: In the logistic regression framework the logit of the probability for a new individual to belong to a target group *p(X)* is modeled through a linear model:

$\log\left(\frac{p(X)}{1-p(X)}\right)=\beta_{0}+\beta_{1}X_{1}+\beta_{2}X_{2}+...+\beta_{p}X_{p}$ Eq. 1

in which $X=\left\{X_{1},X_{2},\ldots,X_{p}\right\}$ contain the *p* predictor values for the individual. If no prior information on class membership is available, the individual is assigned to the target group if *p(X)* > 0.5 (i.e. it has a positive logit).

To build the predictive model of Eq. 1 a training sample of *n* individuals with known group label and known feature values is required. Values of the regression coefficients (betas) maximizing the log-likelihood function (Eq. 2 below) are taken as coefficient estimates.

$LogL=\sum_{i=1}^{n} \left\{I_{i}\left(\beta_{0}+\beta_{1}X_{1i}+\beta_{2}X_{2i}+...+\beta_{p}X_{pi}\right)-\log(1+e^{\left(\beta_{0}+\beta_{1}X_{1i}+\beta_{2}X_{2i}+...+\beta_{1}X_{pi}\right)}\right\}$ Eq.2

where *I_i_* = 1 if the individual belongs to the target class and *I_i_* = 0 if it does not.

In high dimensional settings (as in Figure 1) where the number of features (i.e. number of voxels, cortical vertices or regions) is much larger than the number of individuals (*p* >> *n*) coefficients are not estimable by Eq. 2 alone and further constraints should be applied to the regression estimates. This is done by adding a penalizing (regularizing) term subtracting the log-likelihood value which, for the ridge regression involves the sum of squared coefficients. The maximizing function then becomes

$\max\left\{LogL-\lambda\sum_{j=1}^{p} \beta_{j}^{2}\right\}$ Eq. 3

where regularization strength is modulated by the non-negative parameter lambda chosen through internal cross-validation on the training sample. Squared coefficients in the penalizing function lead to significantly shrunken, but non-null, coefficient estimates. Hence, all predictor variables have some weight in the classification through Eq. 1.

II) **Lasso logistic regression**: Lasso regularization comes from considering the sum of absolute values of the coefficients as an alternative to squaring them [1]. The maximizing function becomes

$\max\left\{LogL-\lambda\sum_{j=1}^{p} \left|\beta_{j}\right|\right\}$. Eq. 4

Taking absolute values leads to a small amount of non-null coefficients (*p*’) and to a sparse solution (with *p*’< *n*) while the remaining variables have no effect on Eq. 1. Lasso regression will be most effective when predictive power is held by a small amount of features.

III) **Elastic net regularization**: This regularization combines both ridge and lasso penalties in the maximizing function [2]

$\max\left\{LogL-\lambda\sum_{j=1}^{p} \left(\frac{1}{2}(1-\alpha) \beta_{j}^{2}+\alpha\left|\beta_{j}\right|\right)\right\}$ Eq. 5

allowing a higher degree of flexibility when fitting Eq. 1. While setting coefficients of all non-informative variables to zero, elastic net regularization assigns a non-null value to variables with individual predictive power. When using images, this leads to selecting neighboring voxels with redundant information but substantial predictive power, generating “clusters” of predictive voxels. Its increased flexibility, though, requires specifying the value of two parameters (lambda and alpha) through internal cross-validation. This involves a two dimensional search, increasing considerably the computational cost in comparison to the ridge and the lasso. We used the glmnet function [3] included in R to fit the ridge, the lasso and the elastic net regressions.

IV) **L0-norm regularization**: This type of regularization imposes a restriction on the number of non-null coefficients in Eq. 1

$\max\left\{LogL-\lambda\sum_{j=1}^{p} I(\beta_{j}\neq0)\right\}$ Eq. 6

where *I* is an indicator variable that equals 1 for non-null coefficients and 0 otherwise. L0-norm regularization, like the lasso, leads to highly sparse models. We applied an adaptation of the multistep adaptive lasso algorithm proposed by [4] to perform the L0 norm regularization.

V) **Support vector classifier (SVC)**: SVC is the linear version of the popular support vector machine. The SVC builds a hyper-plane (linear equation)

$\beta_{0}+\beta_{1}X_{1}+\beta_{2}X_{2}+...+\beta_{p}X_{p}=0$ Eq. 7

that optimally discriminates individuals in the hyper-dimensional feature-space. Individuals located at each side of the hyper-plane are allocated to each of the two groups (as in Eq. 1, subjects with positive values for the left hand side of Eq. 7 are assigned to the target class) and, notably, only training individuals near the discriminating hyper-plane (which are known as support vectors) have some weight in setting Eq. 7.

Although building Eq. 7 from a training sample requires the solution of a complex optimization problem [5] it has been shown that fitting a SVC is equivalent to maximizing

$-1\sum_{i=1}^{n} \max\left[0,1-Y_{i}\left(\beta_{0}+\beta_{1}X_{1i}+\beta_{2}X_{2i}+...+\beta_{p}X_{pi}\right)\right]-\lambda\sum_{j=1}^{p} \beta_{j}^{2}$ Eq.8

where *Y_i_* equals 1 for the target class and -1 for the rest. This formula is similar in structure to Eq. 3, with a second penalizing (regularizing) term involving the sum of squared coefficients. Increasing the weight of this sum with a larger lambda leads to a less restrictive hyper-plane, which allows a larger number of training individuals to be located on the ‘wrong’ side of the plane (in most real applications there is no possible hyper-plane leading to a perfect discrimination of the training sample). For the SVC fittings we applied the computationally efficient LiblinearR function available in R [6].

VI) **Regularized discriminant function analysis (RDA)**: Standard linear discriminant function analysis assumes multivariate normality with common covariance matrix Sigma and a different vector of means (mu) for the individuals belonging to the two groups (g=1,2). This leads to a simple linear discriminant function

$d(X)=\left(X^{T}\Sigma^{-1}\mu_{1}-\frac{1}{2}\mu_{1}^{T}\Sigma^{-1}\mu_{1}\right)-\left(X^{T}\Sigma^{-1}\mu_{2}-\frac{1}{2}\mu_{2}^{T}\Sigma^{-1}\mu_{2}\right)$ Eq. 9

that assigns a new individual to class 1 if its value is positive (and to class 2 if it is negative), where both sigma and mu are initially unknown and are estimated with the training sample. As it happened with the standard logistic regression, when *p* >> *n* (i.e. there are many more features than observations) challenging estimation problems arise.

To avoid them, we have applied the shrunken centroids regularized discriminant function (RDA) analysis [7], which makes Eq. 9 estimable by discarding individual variables with small mean differences (shrinking centroids) and by regularizing the estimation of sigma for the remaining variables. This two-fold process requires adjusting two parameters (alpha and delta) through internal cross-validation, making its implementation more time consuming than the methods involving a single regularizing parameter. The function rda implemented in R has been used to carry out the classifications based on the RDA.

VII) **Gaussian process classifier (GPC)**: Gaussian processes assume that individuals from a given class have a score characterized by a latent (unobservable) variable which quantify the degree of membership to the class. This latent variable is assumed to be normal, and different individuals are assumed to covariate following a covariance function C(*X_i_*, *X_j_*) that depends on their distance in the feature space. By considering a Bayesian framework, the posterior probability of belonging to the target class is calculated [8]. This involves solving a high dimensional integral but, simplifying computationally efficient assumptions are considered in *p* >> *n* situations. For our classifications we used the function gausspr (kernlab package) implemented in R with [9].

VIII) **Random forests (RF)**: Random forests are based on ensembles of classification trees. Single classification trees are built by sequentially partitioning the feature space through successive thresholding (binarization) of individual variables. At each step, the improvement in classification accuracy is evaluated through an index (the Gini index or the cross-entropy) until tree growth stops when no further improvement is achieved. However, classifiers based on single trees are quite unstable (i.e. small changes in the training sample may lead to very different trees). Such instability may be greatly reduced by combining the results from a large set of trees (a random forest), where each tree is generated from a bootstrapped subsample from the training set. Only a random selection of *m* features is considered at each partitioning step of the tree, making branches more de-correlated, and making the random forest classifiers even more stable.

The main drawback of random forests is that the quantitative information of features is lost with binarization. On the other hand, classifying through partitioning the feature space allows fitting much more flexible models which can depart from the additivity restriction imposed by models like those of Eqs. 1, 7 or 9. We have applied the function randomForest implemented in R [10] with *m* = *p*^1/2^. A fixed number of 1000 trees has been set for each fitting.

**References**

1. Tibshirani R (1996) Regression Shrinkage and Selection via the lasso. Journal of the Royal Statistical Society Series B 58: 267–288.

2. Zou H, Hastie T (2005) Regularization and Variable Selection via the Elastic Net. Journal of the Royal Statistical Society, Series B 67: 301–320.

3. Friedman J, Hastie T, Tibshirani R (2010) Regularization Paths for Generalized Linear Models via Coordinate Descent. J Stat Softw 33: 1-22.

4. Bühlmann P, Meier L, van de Geer S (2014) Discussion: A significance test for the lasso. The Annals of Statistics 42: 469–477.

5. Hastie T, Tibshirani R, Friedman JK (2009) The elements of statistical learning. Data mining, inference and prediction. New York: Springer.

6. Helleputte T (2015) LiblineaR: Linear Predictive Models Based On The Liblinear C/C++ Library.R package version 1.94-2.

7. Guo Y, Hastie T, Tibshirani R (2005) Regularized discriminant analysis and its application in microarrays. Biostatistics 1: 1_18.

8. Matthias S (2004) Gaussian Processes for Machine Learning. International Journal of Neural Systems 14: 69-104.

9. Karatzoglou A, Smola A, Hornik K, Zeileis A (2004) kernlab - An S4 Package for Kernel Methods in R. Journal of Statistical Software 11: 1-20.

10. Liaw A, Wiener M (2002) Classification and Regression by randomForest. R News 2: 18-22.
