# Supplementary material for: Evaluation of machine learning algorithms and structural features for optimal MRI-based diagnostic prediction in psychosis
Source: PLoS One. 2017 Apr 20;12(4):e0175683. doi: 10.1371/journal.pone.0175683 (PMC5398548; doi:10.1371/journal.pone.0175683)
Supplement: S2 Appendix — (DOC) [file pone.0175683.s002.doc]

**S2 Appendix. Results from comparing average-over-feature accuracies in the three group pairs.**

Taking classification rates, averaged over features, for each algorithm (see Fig 7 of main document). We compared mean accuracies obtained in the three different group pairs (pair1: healthy vs Schizophrenia; pair2: healthy vs Bipolar disorder; pair3: Bipolar disorder vs Schizophrenia). Specifically, we used non-parametric Wilcoxon (unpaired tests). Below there is a table with mean accuracy values and p-values for the different comparisons.

| pair1 versus pair2 | | | |
| --- | --- | --- | --- |
|  |  |  |  |
| Classifier | mean pair1 | mean pair2 | p-value |
| Ridge | 0.661 | 0.540 | 0.001 |
| Lasso | 0.676 | 0.554 | 0.004 |
| Elastic | 0.678 | 0.544 | 0.002 |
| L0 | 0.650 | 0.542 | 0.001 |
| SVC | 0.656 | 0.551 | 0.000 |
| RDA | 0.681 | 0.552 | 0.000 |
| GPC | 0.668 | 0.549 | 0.002 |
| RF | 0.671 | 0.567 | 0.007 |
|  |  |  |  |
| pair1 versus pair3 | | | |
|  |  |  |  |
| Classifier | mean pair1 | mean pair3 | p-value |
| Ridge | 0.661 | 0.580 | 0.009 |
| Lasso | 0.676 | 0.602 | 0.023 |
| Elastic | 0.678 | 0.605 | 0.043 |
| L0 | 0.650 | 0.553 | 0.005 |
| SVC | 0.656 | 0.572 | 0.005 |
| RDA | 0.681 | 0.590 | 0.006 |
| GPC | 0.668 | 0.602 | 0.029 |
| RF | 0.671 | 0.593 | 0.016 |
|  |  |  |  |
| pair2 versus pair3 | | |  |
|  |  |  |  |
| Classifier | mean pair2 | mean pair3 | p-value |
| Ridge | 0.540 | 0.580 | 0.162 |
| Lasso | 0.554 | 0.602 | 0.026 |
| Elastic | 0.544 | 0.605 | 0.034 |
| L0 | 0.542 | 0.553 | 0.240 |
| SVC | 0.551 | 0.572 | 0.344 |
| RDA | 0.552 | 0.590 | 0.063 |
| GPC | 0.549 | 0.602 | 0.052 |
| RF | 0.567 | 0.593 | 0.450 |

Clearly, average-over-feature accuracies are higher in the healthy vs schizophrenia (pair1) classifications for all different algorithms.
